# How reduction of theta rhythm by medial septum inactivation may covary with disruption of entorhinal grid cell responses due to reduced cholinergic transmission

**DOI:** 10.3389/fncir.2013.00173

**Published:** 2013-10-31

**Authors:** Praveen K. Pilly, Stephen Grossberg

**Affiliations:** ^1^Center for Neural and Emergent Systems, Information and Systems Sciences Laboratory, HRL LaboratoriesMalibu, CA, USA; ^2^Department of Mathematics, Center for Computational Neuroscience and Neural Technology, Center for Adaptive Systems, Boston UniversityBoston, MA, USA

**Keywords:** grid cells, medial entorhinal cortex, self-organizing map, spatial navigation, acetylcholine, oscillations, theta rhythm, medial septum

## Abstract

Oscillations in the coordinated firing of brain neurons have been proposed to play important roles in perception, cognition, attention, learning, navigation, and sensory-motor control. The network theta rhythm has been associated with properties of spatial navigation, as has the firing of entorhinal grid cells and hippocampal place cells. Two recent studies reduced the theta rhythm by inactivating the medial septum (MS) and demonstrated a correlated reduction in the characteristic hexagonal spatial firing patterns of grid cells. These results, along with properties of intrinsic membrane potential oscillations (MPOs) in slice preparations of medial entorhinal cortex (MEC), have been interpreted to support oscillatory interference models of grid cell firing. The current article shows that an alternative self-organizing map (SOM) model of grid cells can explain these data about intrinsic and network oscillations without invoking oscillatory interference. In particular, the adverse effects of MS inactivation on grid cells can be understood in terms of how the concomitant reduction in cholinergic inputs may increase the conductances of leak potassium (K^+^) and slow and medium after-hyperpolarization (sAHP and mAHP) channels. This alternative model can also explain data that are problematic for oscillatory interference models, including how knockout of the HCN1 gene in mice, which flattens the dorsoventral gradient in MPO frequency and resonance frequency, does not affect the development of the grid cell dorsoventral gradient of spatial scales, and how hexagonal grid firing fields in bats can occur even in the absence of theta band modulation. These results demonstrate how models of grid cell self-organization can provide new insights into the relationship between brain learning and oscillatory dynamics.

## Introduction

Medial entorhinal grid cell and hippocampal place cell firing are neural correlates of spatial representation in the brain. While a place cell typically fires whenever an animal is present in a single spatial region, or place, of an environment, each grid cell can fire in multiple spatial regions that form a regular hexagonal grid extending throughout a navigated open field. Neural models have proposed how grid cells of multiple spatial scales can cooperate to activate place cells that can represent much larger spaces than the grid cells can (e.g., Gorchetchnikov and Grossberg, 2007). Since grid cells were reported by Fyhn et al. (2004) and Hafting et al. (2005), a number of neural mechanisms have been proposed to account for their distinctive hexagonal grid spatial firing patterns. They can be broadly classified into three types; namely, oscillatory phase interference, continuous attractors, and self-organizing maps (SOM) (see Zilli, 2012 for a recent review).

For instance, SOM models simulate how grid cell receptive fields may be learned as an animal navigates realistic trajectories (Grossberg and Pilly, 2012; Mhatre et al., 2012; Pilly and Grossberg, 2012, 2013). It is believed that path integration inputs play an important role in activating grid cells (Hafting et al., 2005; McNaughton et al., 2006). Estimates of linear velocity based on path integration activate stripe cells in these models; see Krupic et al. (2012) for data regarding stripe cells. Stripe cells are arranged in rings of cells that are called ring attractor circuits. Different cells in each ring attractor respond at offset spatial positions. Due to the ring structure, each stripe cell responds periodically as its ring attractor integrates linear velocity along a prescribed direction. Multiple stripe cell ring attractors are posited to exist, corresponding to different directions and spatial scales. In response to its ring attractor inputs, the SOM can learn grid cell receptive fields by detecting and amplifying their most frequent and energetic co-activations. Just as stripe cells integrate *linear* velocity, head direction (HD) cells, which encode the direction in which an animal's head is pointed, integrate *angular* velocity. HD cells have typically also been modeled by ring attractors. Thus, both linear velocity and angular velocity are predicted to be processed by homologous ring attractors (Blair et al., 2008; Mhatre et al., 2012).

Oscillatory interference models highlight the possible importance of the theta rhythm in spatial navigation by positing that grid cells are activated by positive interference among neural oscillations whose frequencies are in the theta band (4–11 Hz), are linearly sensitive to running speed, and are selective to movement direction via a cosine tuning function (e.g., Burgess et al., 2007; Hasselmo et al., 2007). In particular, the hexagonal grid correlate of each grid cell's firing is explained by a hardwired combination of a baseline theta oscillation and exactly three active oscillations whose preferred directions differ from each other by 60° and that are in phase (i.e., synchronous) when the animal is present in any one of the grid fields of the cell. In this framework, the spacing and width of grid cell firing fields are inversely proportional to the velocity gain of the oscillation frequencies. Subthreshold membrane potential oscillations (MPOs) observed *in vitro* in MEC layer II stellate cells, whose frequency tends to decrease linearly with location along the dorsoventral axis of MEC (Giocomo et al., 2007); theta rhythm in the local field potential (LFP) of MEC layer II, whose frequency tends to increase with running speed (Jeewajee et al., 2008); and rhythmic bursts of inhibitory “theta cells” in anterior thalamus, hippocampus, and medial septum (MS), whose frequency follows cosine tuning to movement direction (Welday et al., 2011), have been interpreted as evidence for such an oscillatory interference mechanism.

**Figure 1:**
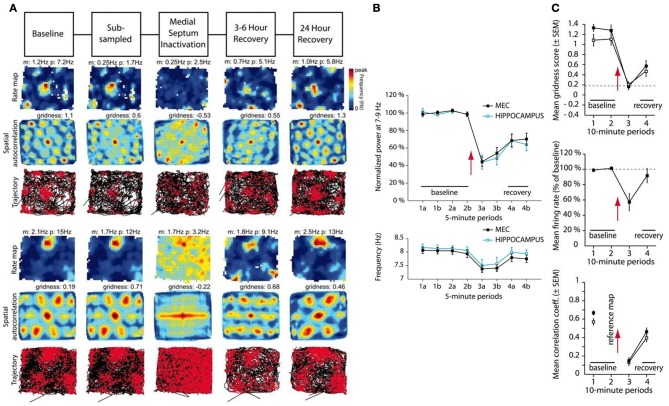
**Data showing effects of medial septum (MS) inactivation on grid cells and network theta oscillations in medial entorhinal cortex (MEC). (A)** Examples of disruption in the spatial expression of the hexagonal grid structure for two grid cells (Brandon et al., 2011). **(B)** Temporal reduction in the power and frequency of network theta oscillations (Koenig et al., 2011). **(C)** Temporary reduction in the gridness score, mean firing rate, and spatial stability of grid cells (Koenig et al., 2011). [Data reprinted with permission from Brandon et al. (2011) and Koenig et al. (2011)].

Recently, Brandon et al. (2011) and Koenig et al. (2011) studied the effects of temporarily inactivating MS using infusions of muscimol and lidocaine, respectively, in dorsal MEC. They found that MS inactivation causes reductions in the power and frequency of MEC network theta oscillations, as well as in the hexagonal gridness quality, spatial stability, and firing rate of grid cells (Figure 1). As the effects of the drugs wash out, the recovery of grid cell properties coincides with that of the theta rhythm. One prominent interpretation of these data has been that the theta rhythm is essential for grid cells to express their spatially periodic firing fields, and thereby that oscillatory interference is indeed at play. Other recent data challenge this view by showing in various ways that the spatial firing fields of grid cells do not depend upon an ongoing theta rhythm (e.g., Yartsev et al., 2011; Killian et al., 2012; Domnisoru et al., 2013; Schmidt-Heiber and Hausser, 2013).

**Figure 2:**
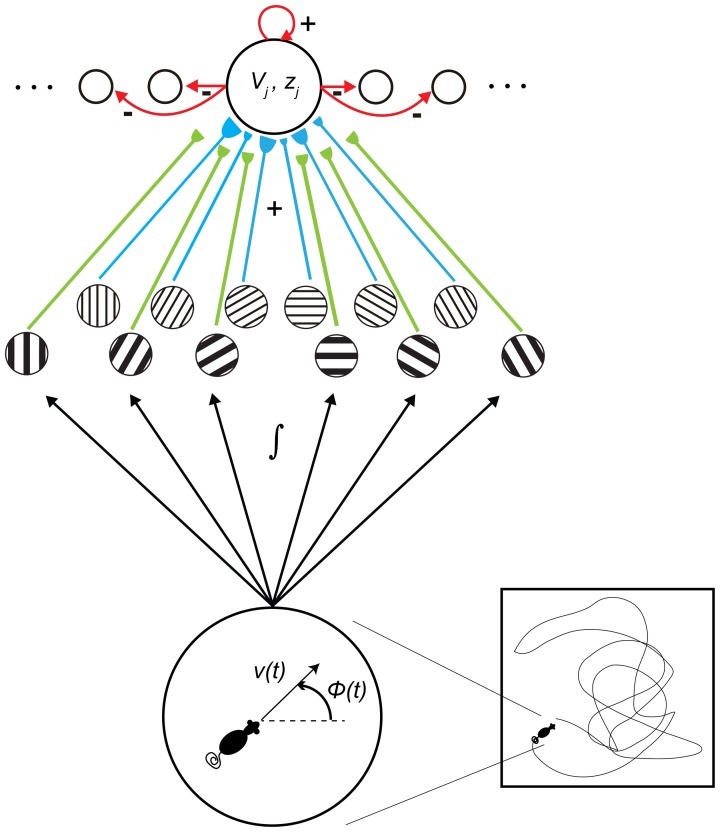
**Macrocircuit of the Spectral Spacing model.** Prior to the development period, entorhinal map cells receive unbiased axonal projections from stripe cells of multiple direction preferences, spatial phases, and spatial scales. Their response rates, or rates of temporal integration, help to select among the input spatial scales of stripe cells during the self-organized learning process that favors the categorical coding of the most frequent and energetic co-active input patterns. [Figure reprinted with permission from Grossberg and Pilly (2012)].

The current article provides an alternative, non-oscillatory account of the MS inactivation data (Brandon et al., 2011; Koenig et al., 2011), based on a SOM model of how grid cell receptive fields are learned during development (Grossberg and Pilly, 2012); see Figure 2. This SOM model has explained and simulated how the gradient of increasing spacing and size of grid cell receptive fields along the dorsoventral axis of MEC (Sargolini et al., 2006; Brun et al., 2008) can be learned as an emergent property of a decrease in cell response rate—that is, in rate of temporal integration (Garden et al., 2008)—along the dorsoventral axis. In particular, in response to inputs of multiple scales from stripe cells, grid cells with faster (slower) response rates can learn to selectively respond to stripe cells with smaller (larger) spatial scales. The kinetics of Ca^2+^-activated K^+^ slow and medium after-hyperpolarization potentials (sAHP and mAHP), which may be controlled by the rate of temporal integration, are proposed to play a critical role in biasing grid cells to learn a particular spatial scale of input stripe cells. Consistently, Navratilova et al. (2012) reported that the recovery time constants of mAHPs are longer for more ventral MEC layer II stellate cells.

This model is called the Spectral Spacing Model due to its ability to select a subset of spatial scales from a spectrum of spatial scales using response rate as a control signal. These multiple-scale grid cells are found in circuits passing through the *medial* entorhinal cortex (MEC) that project to the hippocampus. The term Spectral Spacing emphasizes the homology with an earlier Spectral Timing Model, which clarifies how a subset of temporal scales may be selected from a spectrum of temporal scales, again using response rate as a control signal. These multiple-scale adaptively timed cells are found in circuits passing through the *lateral* entorhinal cortex that project to the hippocampus (Grossberg and Schmajuk, 1989; Grossberg and Merrill, 1992, 1996). These results predict that space and time are both computed in the entorhinal-hippocampal system because their computation is based on a shared circuit design.

The dorsoventral gradient in the rate of temporal integration of MEC layer II stellate cells (Garden et al., 2008) was shown in the Spectral Spacing Model to also account for the gradient in the frequency of subthreshold MPOs (Giocomo et al., 2007; Yoshida et al., 2011), without invoking an oscillatory interference mechanism. This result shows that the frequency of intrinsic MPOs and the spatial scale of the grid fields may not be causally linked, despite being correlated. Such a map-based account is consistent with data that are problematic for oscillatory interference models. Notably, Giocomo et al. (2011) demonstrated that the knockout of the HCN1 gene in mice, which flattens the dorsoventral gradient in MPO frequency and resonance frequency (Giocomo and Hasselmo, 2009), does not affect the development of the grid cell spatial scale gradient. In a similar vein, the current article explains the adverse effects of MS inactivation on grid cells in terms of how a concomitant reduction in cholinergic inputs may increase the conductances of leak potassium (K^+^), and sAHP and mAHP channels, rather than as a result of changes in the theta rhythm *per se*.

## Methods

The MS in the basal forebrain plays an important role in generating and maintaining network theta rhythm in the hippocampal and parahippocampal areas (Vertes and Kocsis, 1997) via reciprocal interactions among GABAergic interneurons (Tóth et al., 1993; Wang, 2002). The MS is also the source of widespread cholinergic projections that target both principal cells and interneurons in these areas via the dorsal fornix. Previous studies have shown that muscimol, which is a GABA_A_-agonist, can inactivate cholinergic cells (Casamenti et al., 1986: nucleus basalis; Vazquez and Baghdoyan, 2004: pontine reticular formation) as well. In addition, there is diminished cholinergic staining in MEC following MS lesions (Mitchell et al., 1982). It is therefore reasonable to assume that injections of muscimol, or lidocaine, into MS result in reduced cholinergic transmission to MEC and hippocampus. Klink and Alonso (1997) reported that application of carbachol, which is a cholinergic agonist, induces slow membrane depolarization and reduces the sAHP current in rat MEC layer II stellate cells, while Müller et al. (1988) showed that these carbachol effects in guinea pig hippocampal CA3 pyramidal cells are blocked by atropine, which is an antagonist of muscarinic acetylcholine receptors (mAChRs). Carbachol also reduces the mAHP current, as observed in hypoglossal motoneurons in rat brainstem (Lape and Nistri, 2000). Further, Madison et al. (1987) reported that increased activation of mAChRs in hippocampal CA1 pyramidal cells enhances their excitability by blocking the leak K^+^ current. In summary, lower-than-baseline levels of acetylcholine (ACh) due to MS inactivation can cause increased conductances of leak K^+^, mAHP, and sAHP currents, thereby slowing the rate of membrane depolarization, and causing longer refractory periods. Within the Spectral Spacing model, the effects of MS inactivation by muscimol, or lidocaine, infusions can therefore be simulated by a temporary reduction in the rate of temporal integration of learned grid cells.

We first simulated the development of two MEC populations using the Spectral Spacing model (Grossberg and Pilly, 2012); see Appendix. This simulation included 50 map cells in each population that received initial random inputs from 72 stripe cells with two spacings (*s*_1_ = 20 cm, *s*_2_ = 35 cm), four spatial phases [*p* = (0, *s*/4, *s*/2, 3*s*/4) for the stripe spacing *s*], and nine movement directions (−80° to 80° in steps of 20°). With an intact MS, the map cells in the two entorhinal populations had response rates (μ_m_) of 1 and 0.6, respectively. Stripe field width was assumed to vary in proportion to stripe spacing. In particular, the standard deviation of the stripe field Gaussian tuning was 8.84% of the stripe spacing (σ_*i*_ = 0.0884 · *s*_*i*_; *i* = 1, 2; see Equation 1.4). Stripe cell peak activity was assumed to be inversely related to spatial scale, along the lines of how the peak firing rate of grid cells decreases with spatial scale (Brun et al., 2008). In particular, the peak activity (ρ_*i*_) was 1 and 0.8 for the stripe spacings *s*_1_ =20 cm and *s*_2_ =35 cm, respectively.

The development of the entorhinal map cells into their adult counterparts was accomplished by employing 20 learning trials, in each of which the model animal ran along a novel realistic trajectory of ~20 min in a circular environment with a radius of 50 cm. These trajectories were obtained by rotating an original rat trajectory (data: Sargolini et al., 2006) about the midpoint of the environment, which is also the starting point, by random angles. The original trajectory was interpolated to increase its temporal resolution to match the time step of numerical integration of model dynamics (Δ*t* = 2 ms). To replicate the reports of Brandon et al. (2011) and Koenig et al. (2011), MS inactivation was invoked indirectly by either a temporary reduction in the cell response rates (μ_*m*_), or a temporary increase in leak conductances (*A*) combined with a temporary decrease in habituation rates (η), for one trial. The recovery of the firing properties was assessed in two succeeding trials with the reinstatement of the normal cellular parameter values (i.e., with the function of MS restored). Seven different cases were simulated to study in detail our hypothesis that it is the reduction in ACh, and not theta rhythm *per se*, that disturbs grid cell firing when MS is inactivated; see Table 1.

**Table 1:** **Details of the various cases that were simulated**.

**Case**	**Parameters during MS inactivation**	**Learning of bottom-up weights during MS inactivation**	**Novel trajectory during MS inactivation**	**Figure(s)**
1	μ_1_: 1 → 0.5;	Yes	Yes	4
	μ_2_: 0.6 → 0.3 [cell response rates are halved]			
2	μ_1_: 1 → 0.25;	Yes	Yes	4–7
	μ_2_: 0.6 → 0.15 [cell response rates are reduced to one-fourth]			
3	μ_1_: 1 → 0.125;	Yes	Yes	4
	μ_2_: 0.6 → 0.075 [cell response rates are reduced to one-eighth]			
4	μ_1_: 1 → 0.25;	No	No	8
	μ_2_: 0.6 → 0.15 [cell response rates are reduced to one-fourth]			
5	*A*: 3 → 3.5; η: 0.05 → 0.0125 [leak conductances increased by 0.5, and habituation rates are reduced to one-fourth]	Yes	Yes	9
6	*A*: 3 → 4; η: 0.05 → 0.00625 [leak conductances increased by 1, and habituation rates are reduced to one-eighth]	Yes	Yes	9
7	*A*: 3 → 3.5; η: 0.05 → 0.00625 [leak conductances increased by 0.5, and habituation rates are reduced to one-eighth]	Yes	Yes	9

## Results

**Figure 3:**
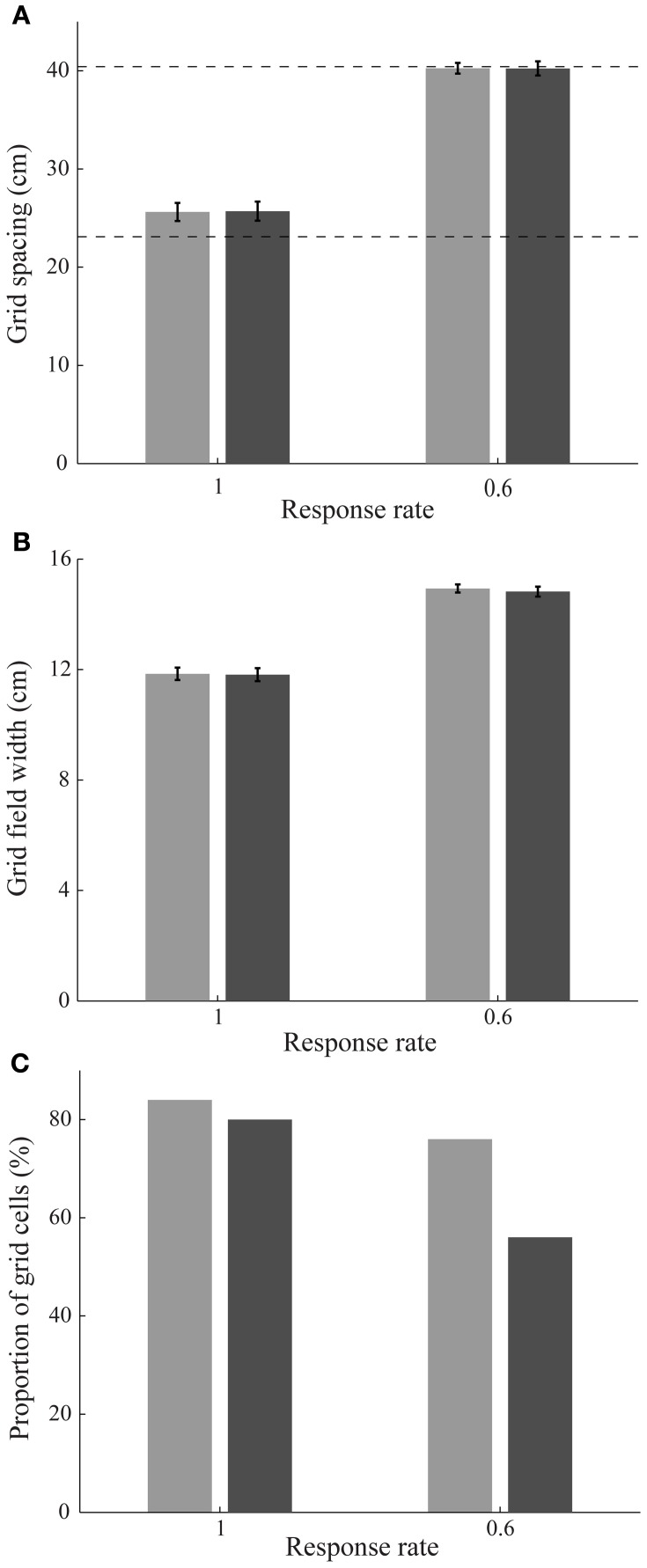
**Stripe cell scale selection depending on entorhinal cell response rates. (A)** Grid spacing, **(B)** grid field width, and **(C)** proportion of learned grid cells in the entorhinal SOMs as a function of response rate at the end of 20 learning trials; see Methods section. Error bars in panels **(A)** and **(B)** indicate standard error of mean (SEM). The light and dark bars correspond to learned grid cells with a gridness score greater than 0 and 0.3, respectively, in the last trial. Dashed horizontal lines in panel **(A)** indicate the two potential grid spacings that the map cells could learn.

**Figure 4:**
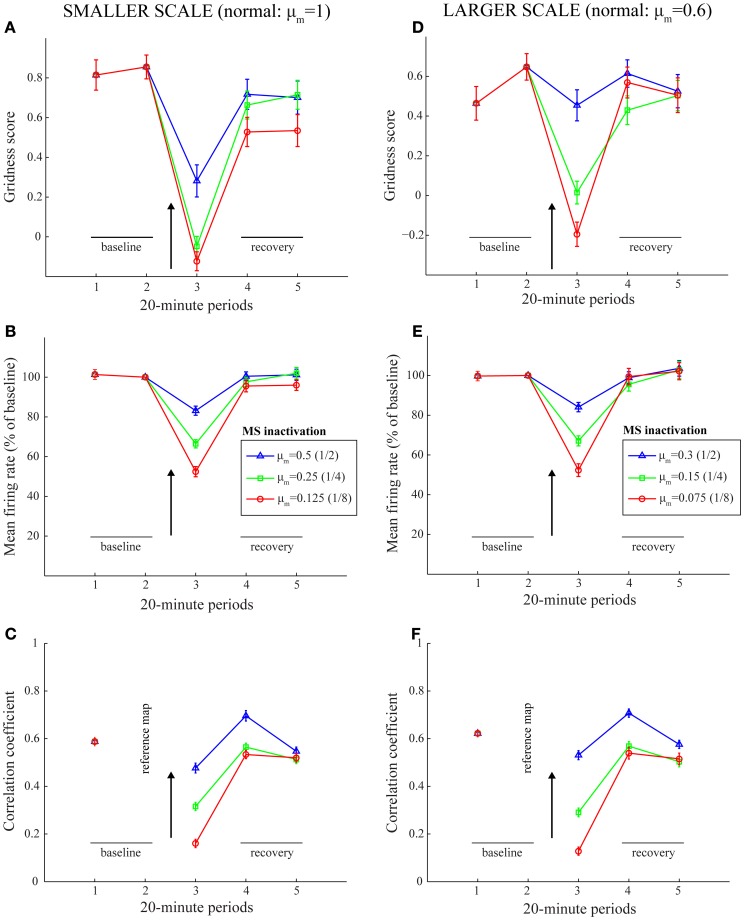
**Model simulation of the MS inactivation data by reduced cholinergic transmission (Cases 1–3).** Simulations of temporary reductions in gridness score **(A,D)**, mean firing rate **(B,E)**, and spatial stability **(C,F)**, respectively, of model grid cells as a result of abrupt changes in cell response rates for one trial. The two columns correspond to the two entorhinal SOMs, which learn to encode two different grid scales of spatial representation. As in panels **(B)** and **(C)** of Figure 1, the arrow in each panel signifies MS inactivation. The legend for the various colored plots is provided in panels **(B)** and **(E)** for the two columns, respectively. The various measures are shown for model grid cells with a gridness score > 0 in the trial immediately preceding the one coinciding with the inactivated MS. Error bars in all panels indicate SEM.

**Figure 5:**
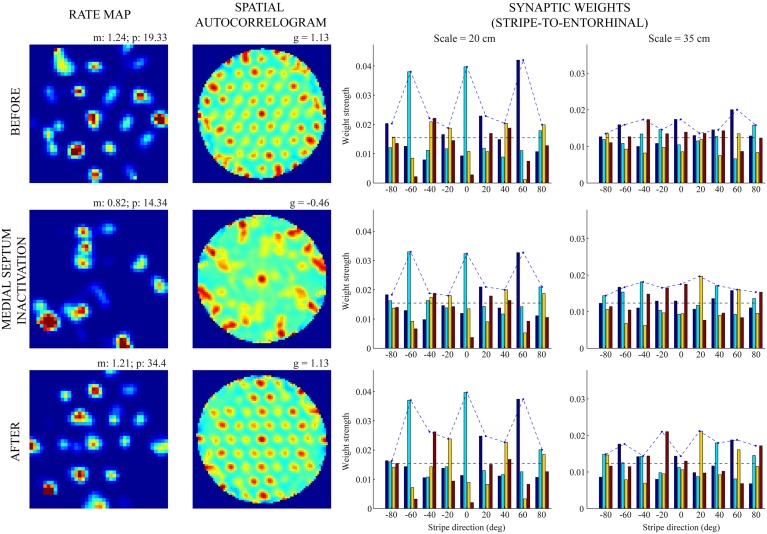
**Spatial responses of a model grid cell with the smaller scale before, during, and after MS inactivation (Case 2).** The rows from top to bottom correspond to three consecutive trials (20th—22nd), with the middle row (21st) being the one in which MS is inactivated. The four columns from left to right show the spatial rate map, its autocorrelogram, weight strengths of connections from stripe cells of the smaller scale (*s*_1_ = 20 cm), and weight strengths of connections from stripe cells of the larger scale (*s*_2_ = 35 cm), respectively, at the end of the trial. Note the mean (m) and peak (p) firing rates, and the gridness score (g) on the top of each rate map and autocorrelogram, respectively. Color coding from blue (min.) to red (max.) is used for each rate map, and from blue (−1) to red (1) for each autocorrelogram.

**Figure 6:**
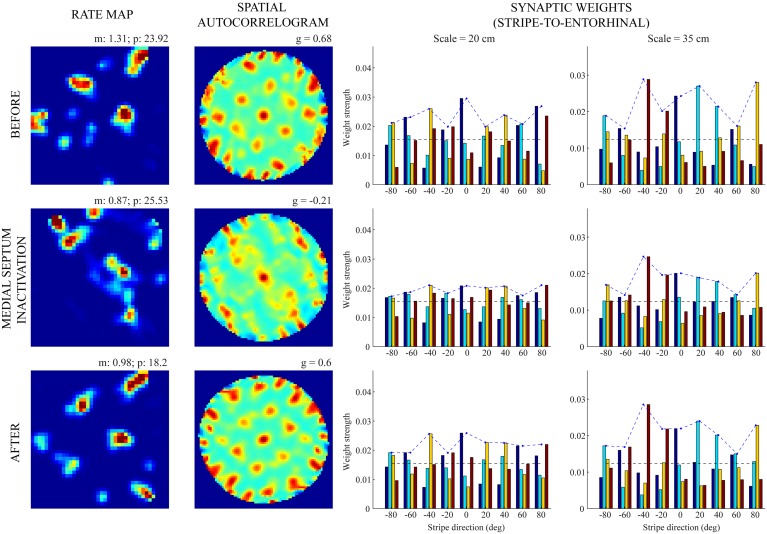
**Spatial responses of a model grid cell with the larger scale before, during, and after MS inactivation (Case 2).** The rows from top to bottom correspond to three consecutive trials (20th—22nd), with the middle row (21st) being the one in which MS is inactivated. The four columns from left to right show the spatial rate map, its autocorrelogram, weight strengths of connections from stripe cells of the smaller scale (*s*_1_ = 20 cm), and weight strengths of connections from stripe cells of the larger scale (*s*_2_ = 35 cm), respectively, at the end of the trial. Note the mean (m) and peak (p) firing rates, and the gridness score (g) on the top of each rate map and autocorrelogram, respectively. Color coding from blue (min.) to red (max.) is used for each rate map, and from blue (−1) to red (1) for each autocorrelogram.

**Figure 7:**
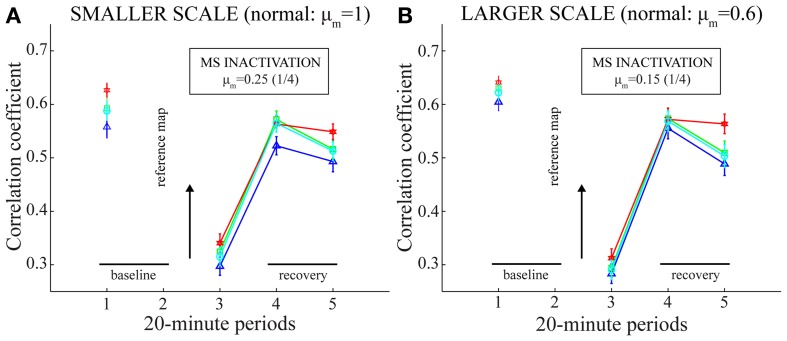
**Spatial stability of model grid cell responses before, during, and after MS inactivation (Case 2).** Same as green plots in panels **(C)** and **(F)** of Figure 4, but with the stability of spatial responses computed in four different ways. Panels **(A)** and **(B)** correspond to the two entorhinal SOMs, respectively. In particular, for a given map cell that has a gridness score > 0 in the baseline trial (i.e., the one before MS is inactivated), the linear correlations between its baseline rate map and its rate maps from pertinent trials are calculated with the consideration of only those spatial bins with a non-zero rate in at least one trial (blue, triangle: Langston et al., 2010; Wills et al., 2010); only those bins with a non-zero rate in both trials (green, square); all bins without any restriction (red, hexagon: Koenig et al., 2011); and only those bins with a non-zero occupancy in both trials (cyan, circle: Brandon et al., 2011).

**Figure 8:**
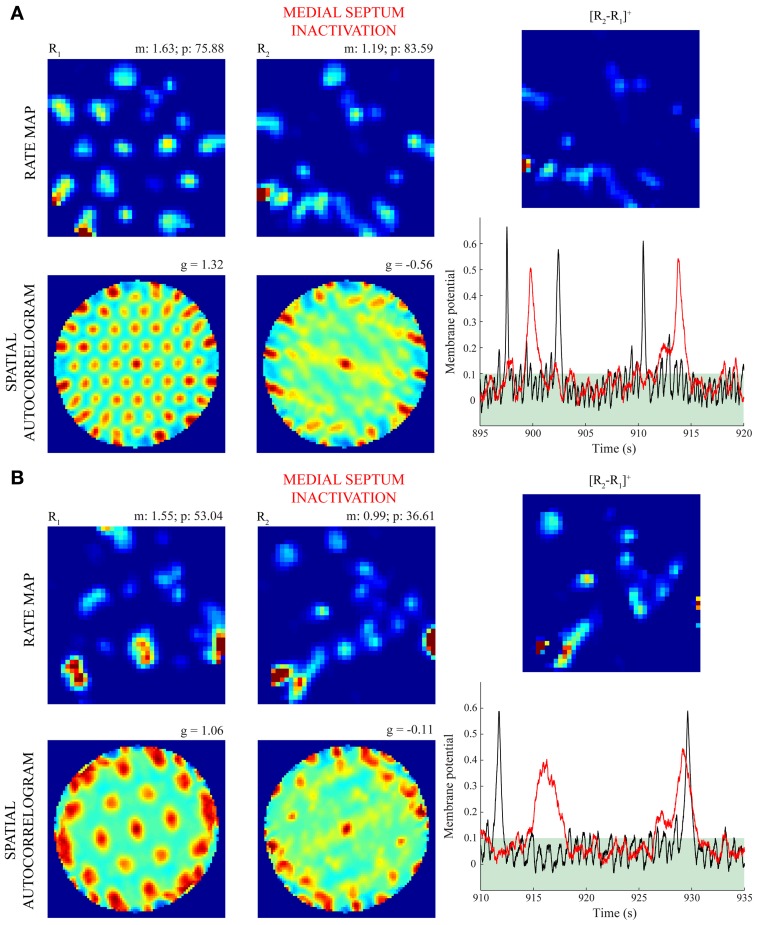
**Firing of model grid cells in non-preferred positions during MS inactivation (Case 4).** Panels **(A)** and **(B)** highlight the differential spatial and temporal responses of two model grid cells with the smaller and larger scales, respectively, between the baseline trial and the inactivation trial. Note for this case the model animal ran along the same realistic trajectory and the bottom-up synaptic weights from stripe cells were not allowed to change (i.e., there was no learning) in either trial. The first two columns show the spatial rate map and autocorrelogram of the cells for these trials. As in Figures 5, 6, the mean (m) and peak (p) firing rates, and the gridness score (g) are provided on the top of each rate map and autocorrelogram, respectively. The top subpanels in the third column show the half-wave rectified differences of the spatial rate maps from the two trials, and the bottom subpanels show the membrane potential dynamics of the cells during 25 s segments through the two trials (black: before; red: during MS inactivation). Note the membrane potential threshold (see Γ in Equations 1.5 and 1.6) of 0.1 for cells to output activity is highlighted in either plot. Color coding from blue (min.) to red (max.) is used for each rate map, and from blue (−1) to red (1) for each autocorrelogram.

**Figure 9:**
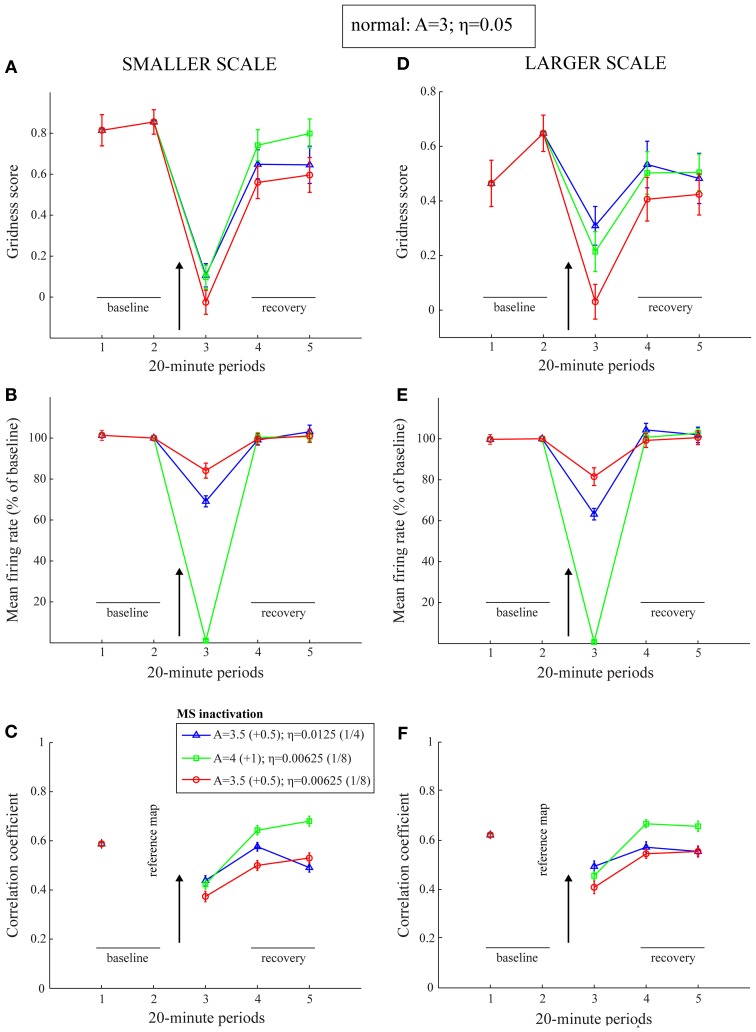
**Model simulation of the MS inactivation data by reduced cholinergic transmission (Cases 5–7).** Simulations of temporary reductions in gridness score **(A,D)**, mean firing rate **(B,E)**, and spatial stability **(C,F)**, respectively, of model grid cells as a result of abrupt changes in leak conductances and habituation rates for one trial. The two columns correspond to the two entorhinal SOMs, which learn to encode two different grid scales of spatial representation. As in panels **(B)** and **(C)** of Figure 1, the arrow in each panel signifies MS inactivation. The legend for the various colored plots is provided in panel **(C)**. The various measures are shown for model grid cells with a gridness score > 0 in the trial immediately preceding the one coinciding with the inactivated MS. Error bars in all panels indicate SEM.

Simulation results are presented in Figures 3–9. We first replicated the main finding of Grossberg and Pilly (2012) that faster response rates (μ_*m*_) of entorhinal map cells cause them to develop hexagonal grid firing fields that are formed from appropriate combinations of stripe cells with the smaller of the input scales, and vice versa (see Figure 3). We then found that temporary reductions in response rates, or rates of temporal integration, can indeed disrupt the expression of learned periodic spatial fields of grid cells by way of delayed and reduced firing with longer refractory periods. Model grid cells are shown to exhibit lower gridness scores, mean firing rates, and spatial stability values during this period in proportion to divisive reductions in response rates; namely, half, one-fourth, and one-eighth (see Figure 4). These results can be understood as direct consequences of reductions in the expected firing for each grid cell at its grid positions due to decreased excitability combined with increased refraction, and also of increased likelihoods for each cell to become activated in non-preferred positions due to lack of expected inhibition from other cells that are activated only weakly if at all.

Figures 5, 6 provide illustrative spatial responses and input synaptic weights of two model grid cells, one each from the two simulated entorhinal SOMs, through the experimental paradigm. Note in either case the distribution of learned connections from input stripe cells, grouped by spatial scale and preferred direction, before MS is inactivated reveals the spatial scale of the hexagonal grid firing field structure that is being encoded. For instance, in the first row of Figure 6, the three stripe cells with the maximal learned weights to the pertinent grid cell share the same larger spacing (namely, *s*_2_ = 35 cm) and have preferred directions of −40°, 20°, and 80°, which are all 60° apart. The erosion of these weights during the period of reduced integration rates occurs with cell firing in spatial positions that do not conform to the encoded grid exemplar (cf. activity-dependent plasticity in Equation 1.6).

The lower spatial stability of model grid cells in the trial coinciding with MS inactivation, compared to the immediately prior one, was ascertained in several ways. Figure 7 confirms this result for four different criteria to include positions, or bins, across the environment in the computation of inter-trial linear correlations of spatial rate maps; namely, regarding (a) only those bins where the firing rate is greater than zero in either trial (Langston et al., 2010; Wills et al., 2010), (b) only those bins where the firing rate is greater than zero in both trials, (c) all bins without any condition (Koenig et al., 2011), and (d) only those bins that were visited by the model animal in both trials (Brandon et al., 2011). To further establish that the decrease in spatial stability is not just due to missing grid firing fields, the model animal was made to run in two trials along the same realistic trajectory and with no further online changes in the strengths of connections from stripe cells to entorhinal map cells. In addition, the second trial involved reductions in cell response rates to one-fourth of their normal values. This allowed for the focused comparisons of spatial and temporal responses of model grid cells between the active and inactive MS conditions. Figure 8 provides observations of two representative model grid cells, one from each of the two entorhinal populations. For either cell, the rectified subtraction of the spatial rate map corresponding to the former trial from that of the latter trial reveals various inconsistent, or non-preferred, positions where the cell became active owing to MS inactivation. This is also clearly apparent in the membrane potential dynamics of the cells between the two trials, with reduced overlap between above-threshold activities.

The period of the inactivated MS has so far been treated in a lumped manner by reduced cell response rates. However, the general trends in the MS inactivation data are also replicated with direct changes in the leak channel and the habituative transmitter gate (*z*^*m*^_*j*_). Figure 9 presents results of lower gridness scores, mean firing rates, and spatial stability values when the leak conductances (*A*) are increased and habituation rates (η) are reduced, with no change in the cell response rates (μ_*m*_). Note that a slower response rate for habituative gating is akin to increasing the conductances of AHP channels. This is because the habituative gate in the model is a phenomenological variable that regulates the duration of the refractory period by multiplicatively gating the critical self-excitatory conductances (see Equation 1.5). Increased leak conductances contribute to reduced and delayed firing of entorhinal map cells.

## Discussion

This article contributes to the ongoing debate on the role of the theta rhythm in key brain areas involved in spatial learning and memory. In this regard, its main contribution is to advance a principled alternative explanation for the adverse effects of MS inactivation on entorhinal grid cells (Brandon et al., 2011; Koenig et al., 2011). This article suggests that factors other than the reduction of theta rhythm *per se* during MS inactivation may cause grid cells to lose spatial periodicity and exhibit lower stability and firing rates. Model simulations and experimental data suggest an important role for reduced cholinergic release and concomitant changes in leak K^+^, mAHP, and sAHP currents. These results are consistent with the explanation by the model of many data about the development and neurophysiology of grid cells and place cells during normal conditions (Pilly and Grossberg, 2012). In this regard, *in vivo* observations that cholinergic neurons in MS, compared to GABAergic neurons, have low firing rates that vary on slow time scales of several tens of seconds (Zhang et al., 2010) are not inconsistent with this conclusion, because grid cell recordings ensued 5 min after the start of lidocaine infusions into MS for the Koenig et al. (2011) study, and 2 min after the start of muscimol infusions into MS for the Brandon et al. (2011) study. Ongoing modeling is starting to characterize how ACh-dependent modulations of various ion channels, notably slow, medium, and fast AHP currents, can influence biophysical properties of individual spiking neurons (Palma et al., 2012b) and networks of such neurons (Palma et al., 2012a). Such studies may in the future be applied to a spiking network model of grid cells (Pilly and Grossberg, 2013) to more completely analyze the disruptive effects on grid cells of silencing the MS. This study can also include the relative contributions of other modulated ion channels in MEC layer II stellate cells, such as the HCN1 channel (e.g., Heys and Hasselmo, 2012; Tsuno et al., 2013) and the m-channel (e.g., Heys et al., 2010).

The Spectral Spacing model (Figure 2; Grossberg and Pilly, 2012) proposes that putative grid cells, during the development period, become tuned to respond preferentially to different favored combinations of co-active stripe cells based on their response rates, or rates of temporal integration. In particular, dorsal cells with faster response rates, and thereby shorter refractory periods, self-organize to be primarily driven by triplets of co-active stripe cells with a smaller spacing and whose preferred directions are separated by 60°. The slower response rates of ventral cells cause them to be controlled by similar triplets of stripe cells with a larger spacing. This can be intuitively understood as follows: (a) Despite non-stationary variations in running speed and heading direction during realistic navigation in an open field, the average time interval between two consecutive firing episodes of an adult grid cell is directly proportional to its grid spacing. (b) Among various co-active stripe cell triplets of compatible spatial scales, the ones with the smallest scale are selected because of their higher frequency of occurrence in two-dimensional space. (c) Because the synapses of axonal projections from stripe cells undergo activity-dependent self-normalized learning (see Equation 1.6), connections learned by the favored spatial scales bias against control by co-active stripe cell inputs that recur, on average, with a time interval that is smaller than the developing grid cell's relative refractory period. Given these properties, if the MS is temporarily inactivated during adulthood, the sudden reduction in overall cell excitability does not allow the affected grid cells to immediately display a new hexagonal grid structure of a larger spacing in their spatial responses.

Brandon et al. (2011) also reported that HD cells and conjunctive (grid × HD) cells in the MEC maintain their directional tuning during the period of MS inactivation, although conjunctive cells lost their grid firing fields. Koenig et al. (2011) showed, in addition, that the firing rate of HD cells does not undergo any significant change. These data suggest that neurons that code HD are not affected by low ACh levels. Given the proposal that stripe cells are implemented using ring attractor circuits that are homologous to those of HD cells (Grossberg and Pilly, 2012; Mhatre et al., 2012; Pilly and Grossberg, 2012), we predict that stripe cells (Krupic et al., 2012) as well may not be greatly impacted as a result of inactivating the MS. Presumably, HD cells and stripe cells either do not express mAChRs, or do not have leak K^+^, mAHP, and sAHP channels. Observed disruptions in periodic spatial firing of grid cells are thus not due to changes in their inputs, but due to changes in their excitability and, along with it, changes in learned network interactions at different spatial positions.

Koenig et al. (2011) also examined the effects of inactivating MS on hippocampal place cells, and found that they largely maintain their place firing fields, but show reductions in firing rate and theta band modulation. This provides additional support to our model's prediction that the theta rhythm is not crucial for medial entorhinal-hippocampal cells to encode spatial information. Longer refractory periods that result from reduced cholinergic action do not adversely affect place cells because they do not have the multiple periodic spatial fields of grid cells and, in addition to grid cell inputs, they also receive reliable sensory, notably visual, inputs in a familiar environment.

Other recent data also support the view that mechanisms other than theta band modulation give rise to spatial properties of grid cells. For example, Yartsev et al. (2011) showed that hexagonal grid firing fields in crawling bats can occur even in the absence of theta band modulation of spiking, and of continuous theta rhythm in the LFP. An additional problem for oscillatory interference model variants in which baseline oscillation frequency does not change through time (e.g., Burgess et al., 2007) is that they can generate hexagonal grids even when the baseline oscillation frequency is set to zero, which is not consistent with the MS inactivation data.

A subclass of continuous attractor network (CAN) models (Fuhs and Touretzky, 2006; Burak and Fiete, 2009) has been suggested as the most consistent among existing grid cell models with recent experimental evidence (Couey et al., 2013; Domnisoru et al., 2013; Schmidt-Heiber and Hausser, 2013). For example, Couey et al. (2013) and Pastoll et al. (2013) verified that stellate cells in layer II of MEC interact with each other not via recurrent excitatory connections but primarily through disynaptic inhibition. Also, Domnisoru et al. (2013) and Schmidt-Heiber and Hausser (2013) used *in vivo* whole-cell recordings during virtual reality navigation to conclude that the spatial field-selective firing of grid cells is better explained by membrane potential ramps caused by integration of synaptic inputs on a slower, sub-theta time scale, and not by constructive interference among intrinsic MPOs in the theta band.

Note the above mentioned data constraints are also consistent with the SOM family of models (Grossberg and Pilly, 2012; Mhatre et al., 2012; Pilly and Grossberg, 2012, 2013). In particular, entorhinal map cells in the SOM models interact in a recurrent inhibitory network. And unlike the CAN models, neighboring grid cells can have spatial fields that are uncorrelated to their anatomical arrangement (Hafting et al., 2005). Further, SOM model grid cells develop such that their membrane potential dynamics respond in a graded fashion to the degree of co-activations among their respective preferred synaptic inputs (see Figures 15a,d in Pilly and Grossberg, 2013). The relatively faster theta oscillations in the in-field membrane potential dynamics of grid cells (Domnisoru et al., 2013; Schmidt-Heiber and Hausser, 2013), which correlate strongly with spike timings, could be modeled as the effect of more synchronous excitatory synaptic currents in the presence of non-specific rhythmic inhibition from interneurons such as theta cells. Similarly, the temporal coding property of theta phase precession seen in grid cells (Hafting et al., 2008) could be understood as resulting from an interaction between increasing excitatory synaptic currents during spatial field traversals and activity-dependent inhibitory currents such as AHP currents. There is now a growing understanding that temporal coding does not principally determine the formation of the space code in the entorhinal-hippocampal system (cf. Harvey et al., 2009; Domnisoru et al., 2013; Schmidt-Heiber and Hausser, 2013). Consistently, Grossberg and Pilly (2012) showed that the subthreshold MPOs of MEC layer II stellate cells, which are generally cited in support of oscillatory interference models (e.g., Burgess et al., 2007; Giocomo et al., 2007; Yoshida et al., 2011), are an emergent property resulting from interactions among cellular components that are needed for distributed map learning in response to different input patterns.

Overall, the results presented in this article combined with those from our earlier work (Grossberg and Pilly, 2012) explain how intrinsic and network oscillations that correlate with characteristic spatial coding properties of grid cells may not play a causal role in their generation, and thus how mechanisms of grid development and learning may explain data that are difficult for oscillatory interference models to accommodate. The current SOM modeling framework can be extended to also clarify how other types of oscillations may occur (Grossberg, 2009), such as the hippocampal beta oscillations that have been reported during initial spatial learning in novel environments (Berke et al., 2008), and the gamma oscillations that are predicted to be correlated with the dynamic stabilization of place cell spatial learning by top-down attentional mechanisms (e.g., Morris and Frey, 1997; Kentros et al., 2004). Taken together, these results illustrate the importance of models of spatial navigation that are capable of self-organizing their spatial representations as an animal navigates around its environment.

### Conflict of interest statement

The authors declare that the research was conducted in the absence of any commercial or financial relationships that could be construed as a potential conflict of interest.

## Appendix

This section describes the Spectral Spacing model equations (Grossberg and Pilly, 2012) that were used in the MS inactivation simulations. Model parameters that were not mentioned in the Methods are provided in Table A1.

**Table A1:** **Model parameters**.

**A**	**B**	**C**	**α**	**β**	**γ**	**λ**	**μ**	**Γ**
3	1	0.5	17.5	1.5	0.2	0.025	0.05	0.1

### Stripe cells

Stripe cells with different spatial phases integrate linear velocity along multiple directions in ring attractor circuits of various spatial scales. They are algorithmically computed, for simplicity, as follows: If at time *t* the animat heads along allocentric direction φ (*t*) with velocity *v* (*t*), then the velocity *v*_*d*_ (*t*) along direction *d* is:

(1.1) $$v_{d}\;\left(t\right)=\cos\;\left(d-\varphi \left(t\right)\right)\;v\left(t\right).$$

The displacement *D*_*d*_ (*t*) traversed along direction *d* with respect to the initial position is calculated by path integration of the corresponding velocity:

(1.2) $$D_{d}\;\left(t\right)=\int_{0}^{t}v_{d}\;\left(\tau \right)\;d\tau .$$

This directional displacement variable is converted into activations of stripe cells that prefer different spatial phases *p* along a ring attractor that is selectively tuned to direction *d* and spatial scale *s*. Let *x*_dps_ (*t*) be the activity of a stripe cell whose spatial fields are oriented perpendicular to direction *d* with spatial phase *p* and spatial period *s*. This stripe cell has maximal activity at periodic positions *ns* + *p* along direction *d*, for all integer values of *n*. Activity *x*_dps_ (*t*) will thus be maximal whenever (*D*_*d*_ modulo *s*) = *p*, where the modulo operator computes the remainder when *D*_*d*_ is divided by *s*, and thus resets the displacement modulo the period *s*. This periodically reset displacement, computed with respect to spatial phase *p* is:

(1.3) $$D_{\text{dps}}\;\left(t\right)=\left(D_{d}\;\left(t\right)-p\right)\text{modulo }s.$$

Thus, if the stripe cell *x*_dps_(*t*) has a Gaussian-like spatial firing profile, then its activity can be modeled as:

(1.4) $$x_{\text{dps}}\left(t\right)=\rho_{s}\cdot \exp\;\left(-\frac{{\left(\min\left(D_{\text{dps}}\;\left(t\right),s-D_{\text{dps}}\left(t\right)\right)\right)}^{2}}{2\sigma_{s}^{2}}\right)\;,$$

where ρ_*s*_ is the maximal activity and σ_*s*_ is the standard deviation of each of its individual stripe fields along the direction *d*. The simulations were carried out with two, or three, spatial scales *s* of stripe cells converging on individual category cells. Learning determines which stripe cell spatial scale gains control of each category cell through time, and how that results in its learned grid scale. Simulations demonstrate how the response rate of a category cell determines its learned grid scale. The directional displacement variables *D*_*d*_(*t*) were all initialized to 0 at the start of each trial.

### Category cells

The membrane potential *V*^*m*^_*j*_ of the MEC layer II category cell *j* in the dorsal population *m* obeys membrane equation, or shunting, dynamics within a recurrent on-center off-surround network (Grossberg, 1976, 1980) as follows:

(1.5) $$\begin{array}{l} \frac{dV_{j}^{m}}{dt}=10\mu_{m}\;[-AV_{j}^{m}+\left(B-V_{j}^{m}\right)\\ \;\left(\sum_{\text{dps}}w_{\text{dpsj}}^{m}x_{\text{dps}}+\alpha \;{\left({\left[V_{j}^{m}\right]}^{+}\right)}^{2}z_{j}^{m}\right)\\ \;-\left(C+V_{j}^{m}\right)\;\sum_{k\;\neq \;j}\beta \;{\left({\left[V_{k}^{m}-\Gamma \right]}^{+}\right)}^{2}]\;, \end{array}$$

where μ_*m*_ controls the rate of temporal integration of the cell (called the response rate); *A* is the decay parameter corresponding to the leak conductance; *B* and −*C* are the reversal potentials of the excitatory and inhibitory channels, respectively; *w*^*m*^_dpsj_ is the synaptic weight of the projection from the stripe cell with activity *x*_dps_ in Equation 1.4 to the category cell *j* in population *m*; α ([*V*^*m*^_*j*_]^+^)^2^ is the on-center self-excitatory feedback signal of the cell, which helps to resolve the competition among category cells within cell population *m*, where [*V*]^+^ = max (*V*, 0) defines a threshold-linear function, and α is the gain coefficient; *z*^*m*^_*j*_ is the habituative transmitter gate of category cell *j*; and β is the connection strength of the inhibitory signal ([*V*^*m*^_*k*_ − Γ]^+^)^2^ from category cell *k* in the off-surround to category cell *j* within population *m*. The output activity of category cell *j* is given by ([*V*^*m*^_*j*_ − Γ]^+^)^2^, which is the same as its recurrent inhibitory signal to other cells in the population. The membrane potential of each category cell was initialized to 0 at the start of each trial.

### Adaptive weights

The adaptive weights *w*^*m*^_dpsj_ of projections from stripe cells to category cells are governed by a variant of the competitive instar learning law (Grossberg, 1976; Grossberg and Seitz, 2003):

(1.6) $$\begin{array}{l} \frac{dw_{\text{dpsj}}^{m}}{dt}=\lambda \;{\left({\left[V_{j}^{m}-\Gamma \right]}^{+}\right)}^{2}\\ \;\left[\left(1-w_{\text{dpsj}}^{m}\right)x_{\text{dps}}-w_{\text{dpsj}}^{m}\sum_{\left(p,\;q,\;r\right)\;\neq \;\left(d,\;p,\;s\right)}x_{\text{pqr}}\right]\;, \end{array}$$

where λ is the learning rate; the category cell output signal ([*V*^*m*^_*j*_ − Γ]^+^)^2^ gates learning on and off; and the learning rule defines a self-normalizing competition among afferent synaptic weights to the target cell, leading to a maximum learned total weight to the cell of 1. Each weight *w*^*m*^_dpsj_ was initialized to a random value drawn from a uniform distribution between 0 and 0.1 at the start of the first learning trial. Equation 1.6 can be rewritten with term [(1 − *w*^*m*^_dpsj_) *x*_dps_ − *w*^*m*^_dpsj_ ∑ _(*p, q, r*) ≠ (*d, p, s*)_ *x*_pqr_] replaced by (*x*_dps_ − *w*^*m*^_dpsj_ ∑ _(*p, q, r*)_ *x*_pqr_), which shows that the weight *w*^*m*^_dpsj_ is attracted to a time-average of the ratio of input activities during the times when the gating, or learning, signal ([*V*^*m*^_*j*_ − Γ]^+^)^2^ is positive. This fact embodies the intuition that the learning law conserves the total number of synaptic learning sites at each map cell by a homeostatic combination of excitatory and inhibitory influences.

### Habituative gating

The habituative transmitter *z*^*m*^_*j*_ of category cell *j* in population *m* is defined by:

(1.7) $$\frac{dz_{j}^{m}}{dt}=10\eta \;\left[\left(1-z_{j}^{m}\right)-\gamma z_{j}^{m}{\left(\alpha \;{\left({\left[V_{j}^{m}\right]}^{+}\right)}^{2}\right)}^{2}\right],$$

where η controls the overall response rate of the transmitter (called the habituation rate) and γ modulates its depletion rate. In particular, term (1 − *z*^*m*^_*j*_) controls the gate recovery rate to the target level of 1, and term −γ *z*^*m*^_*j*_ (α([*V*^*m*^_*j*_]^+^)^2^)^2^ controls the gate inactivation rate, which is proportional to the current gate strength *z*_*j*_ times the square of the signal (α([*V*^*m*^_*j*_]^+^)^2^) that *z*^*m*^_*j*_ gates in Equation 1.5. The squaring operation causes the gated signal to first increase and then decrease through time in response to excitatory input (cf. Gaudiano and Grossberg, 1991), thereby regulating the duration of intense cell activity, and thus cell perseveration. The habituative transmitter of each category cell was initialized to its maximum value of 1 at the start of each trial.

## Post-processing

The 100 × 100 cm environment was divided into 2.5 × 2.5 cm bins. During each trial, the amount of time spent by the model animal in the various spatial bins was tracked. The output activity of each category cell in every spatial bin was accumulated as the trajectory visited that bin. The occupancy and activity maps were smoothed using a 5 × 5 Gaussian kernel with standard deviation equal to one. At the end of each trial, smoothed and unsmoothed rate maps for each category cell were obtained by dividing the cumulative activity variable by cumulative occupancy variable in each bin. Peak and mean firing rates for a category cell in a given trial were obtained by considering all spatial bins in the corresponding smoothed rate map. For each category cell, six local maxima with *r* > 0.05 and closest to the central peak in the spatial autocorrelogram of its smoothed rate map were identified. Gridness score, related to rotational symmetry, was then derived using the method described in Wills et al. (2010). Spatial stability of each category cell in the 19th, 21st (MS inactivation), 22nd, and 23rd trials was defined in reference to the 20th trial as the Pearson's linear correlation coefficient between its smoothed rate maps from those trials and the 20th trial, considering only those bins with occupancy greater than zero in both trials (Brandon et al., 2011).
